# In silico and structural analysis of *Bacillus licheniformis* FAO.CP7 pullulanase isolated from cocoa (*Theobroma cacao* L.) pod waste

**DOI:** 10.1186/s12866-025-03958-w

**Published:** 2025-04-30

**Authors:** Frank Abimbola Ogundolie, Tolulope Peter Saliu, Michael Obinna Okpara, Jacqueline Manjia Njikam, Folasade Mayowa Olajuyigbe, Joshua Oluwafemi Ajele, Gattupalli Naresh Kumar

**Affiliations:** 1 Enzymology and Enzyme Technology Unit, Department of Biochemistry, Federal University of Technology, Akure, Nigeria; 2 Computation and Molecular Biology Unit, Department of Biochemistry, Federal University of Technology, Akure, Nigeria; 3 Enzyme Biotechnology and Environmental Health Unit, Department of Biochemistry, Federal University of Technology, Akure, Nigeria; 4 Microbial and Molecular Biology Laboratory, Department of Biochemistry, Maharaja Sayajirao University of Baroda, Vadodara, India; 5 Department of Biotechnology, Faculty of Computing and Applied Sciences, Baze University, Abuja, Nigeria; 6 Department of Biochemistry, Faculty of Science, University of Yaounde´ I, Yaounde´, Cameroon; 7 Biomedical Biotechnology Research Unit (BioBRU), Department of Biochemistry, Microbiology, and Bioinformatics, Rhodes University, Grahamstown, South Africa; 8 Department of Physiology, College of Medcine, University of Kentucky, Kentucky, USA

**Keywords:** Pullulanase, *Bacillus licheniformis*, Enzymes, Phylogenetic analysis, Bioinformatics, Cocoa pod waste

## Abstract

Pullulanase (EC 3.2.1.41) is an important debranching enzyme that plays a critical role in maximizing the abundant energy present in branched polysaccharides. Its unique ability to efficiently degrade branched polysaccharides makes it crucial in industries like biofuels, food, and pharmaceuticals. Therefore, discovering microbes that produce pullulanase and thrive in harsh industrial conditions holds significant potential for optimizing large-scale bioprocessing. This unique property has made pullulanase an important enzyme in the industry. Thus, the search for microbes that have the pullulanase production properties and capacity to withstand harsh industrial conditions will be of high industrial relevance. Therefore, this study aimed to amplify, sequence, and molecularly characterize the pullulanase gene encoding extracellular pullulanase in *Bacillus licheniformis* strain FAO.CP7 (Accession No: MN150530.1.) which was obtained from cocoa pods using several bioinformatics tools. The amplified *PulA* gene had a nucleotide sequence of 2247 base pairs encoding a full-length open reading frame (ORF) pullulanase protein of 748 amino-acids residues with molecular weight 82.39 kDa and theoretical isoelectric point of 6.47, respectively. The deduced pullulanase protein had an aliphatic index of 77.66. Using BLASTp, the deduced amino acid sequence of the pullulanase gene showed 85% homologies with those from *B. licheniformis* strains. Multiple sequence alignment of PulA protein sequence showed that it contains YNWGYNP motif which is also found in all type I pullulanase protein sequences analysed. The restriction mapping of the gene showed that it can be digested with several restriction enzymes. Further analysis revealed that the deduced protein had a hydrophobicity score of − 0.37 without a transmembrane helix. Overall, this study revealed the *PulA* gene of *B. licheniformis* strain FAO.CP7 obtained from cocoa pods and its deduced protein show significant potential for enhancing starch bioprocessing. With further optimization, it could offer substantial benefits to starch-based biotechnological industries.

**Supplementary Information:**

The online version contains supplementary material available at 10.1186/s12866-025-03958-w.

## Introduction

Pullulanases (EC 3.2.1.41) are a member of the glycoside hydrolase 13 (GH13) enzymes family that catalyze the hydrolysis of α-1,6-glycosidic bond in starch, amylopectin, pullulan, and related oligosaccharides [1, 2]. The α-1, 6 glycosidic linkages in branched polysaccharides serve as a barrier for maximizing the abundant energy present in branched polysaccharides during the saccharification process. Pullulanase are required during the Saccharification process of industrial glucose production to increase the final concentration of glucose [3]. Due to this important feature, pullulanase have attracted attention and found wide applications in starch processing, brewing, detergent, textile, and pharmaceutical industries [3–5]. Based on their substrate specificity and reaction products, pullulanase can be divided into two classes: type I (neopullulanase) and type II (isopullulanase). Though type I and type II pullulanase have the potential to break α-1,6 glycosidic linkages, only type II pullulanases can break α-1,4 glycosidic linkages [3]. Thus, pullulanases are very essential in starch processing and other related industries where they are used to improve the quality and yield of glucose syrup and beer [2].

Starch processing involves harsh conditions such as acidic pH (pH 4.5) and high temperatures ranging from 50–60 °C [6]. Consequently, there is a need to produce pullulanase enzymes that are stable and active under such harsh starch processing conditions. Microbial sources of enzymes are the best for the production of enzymes that can remain stable and active under harsh conditions [7]. Pullulanases have been expressed from various microbial sources such as *Pichia pastoris* [8], *Bacillus* sp. AV-7 [9], *Anoxybacillus* sp. SK3-4 [10], *Escherichia coli* [11–13], *Thermus thermophiles* [14], and *Bacillus subtilis* [15, 16] using different growth substrates.

Cocoa bean pulp and its waste by-product cocoa pod have been shown to have a pH range of 3.3–6.0 due to high citric acid concentration [17]. They are also rich in nutrients that support the growth and proliferation of various microbes [18]. Therefore, it will be industrially relevant to search for microbes that have the pullulanase production properties from this source.

*Bacillus licheniformis* is a non-pathogenic and non-toxigenic microbe that is closely linked to the degradation of nutrient material present in cocoa bean pulp during fermentation [19, 20]. Cocoa bean pulp has been known to be rich with high content of sugars and other organic materials*.* These organic materials favour the growth of *B. licheniformis* which becomes predominant in the later stages of its fermentation and is involved in degrading the pulp for development of various by-products [19, 21]. *Bacillus licheniformis* also derives essential nutrients from cocoa pod husk (CPH) by secreting multiple starch-hydrolysing enzymes, especially pullulanase, which degrade the starch present in CPH [18, 22]. Thus, this study aimed to amplify, sequence, and characterize the pullulanase gene of *B. licheniformis* strain FAO.CP7 obtained from cocoa pod waste using in silico analyses.

## Materials and methods

### Sample collection

The Cocoa pod husk (CPH) used in this study were collected from a cocoa dumpsite in Ajebambo Farms, Longitude (E 5^0^5′50) Latitude (N 7^0^12′44) in Idanre Local Government Area of Ondo State, one of the leading cocoa producing cities in South-Western Nigeria. The CPH were chopped to pieces using a sterile knife and sundried until a constant weight was achieved. The dried CPH were ground and sieved using 0.075 to 1.00 mm mesh sizes to achieve uniform sizes. The sieved samples were stored at 4 °C till further usage.

### Isolation and identification of *B. licheniformis* strain FAO.CP7

Starch utilizing *Bacillus spp.* was isolated from cocoa pod using modified nutrient agar (pulverized cocoa pod (10 g/L), sodium chloride (5 g/L), and agar–agar (20 g/L) with the addition of nystatin (10 mg/mL) to prevent fungal contamination. The selected strain was identified and classified primarily through morphological observation and biochemical tests including starch hydrolysis, indole production, catalase, urease activity, citrate utilization test (CU), gelatin hydrolysis, nitrate reduction test, Voges- Proskauer test (VP), methyl red test (MR), oxidase test, utilization of D-glucose, D-lactose, D-xylose, sucrose, starch, arabinose, maltose, and fructose (see supplementary for methods). For molecular identification of the isolate, the strain was subjected to genomic DNA isolation, 16S rRNA amplification, and sequencing [23].

### Genomic DNA isolation

The modified cetyl trimethylammonium bromide (CTAB) method [24] was used to extract genomic DNA from *B. licheniformis* strain FAO.CP7. The integrity of the isolated DNA was verified by gel electrophoresis using 1% agarose gel stained with ethidium bromide (5 μg/μL). Bio-Rad Gel Documentation system (Bio-Rad, USA) was used to capture the images of bands.

### PCR amplification of 16S rRNA gene

The bacterial 16S rRNA was amplified using the oligonucleotide primers, 27F- 5’-AGAGTTGATCCTGGCTCAG-3’ and 1492R- 5’-TACGGYTACCTTGTTACGACTT-3’ (Except otherwise stated, primers used in this study were obtained from Integrated DNA Technology, USA). The PCR reaction mix contained 1 μL of genomic DNA as a template, 3 μL of forward primer, 3 μL of reverse primer, 4 μL of dNTPs (2.5 mM each), 10 μL of 10X Taq DNA polymerase assay buffer, 1 μL of Taq DNA polymerase enzyme (3 U/μL) and reverse osmosis purified water was added to make up a total reaction volume of 24 μL. PCR cycling parameters were an initial denaturation step (94 °C, 5 min), 35 cycles of denaturation (94 °C, 30 s), annealing (55 °C, 30 s), an initial extension step (72 °C, 90 s) and a final extension step (72 °C, 5 min). The amplified products were analysed on 1% agarose gel electrophoresis in TAE buffer [23].

### Sequencing and bioinformatic analysis of 16S rRNA gene

The amplified products were purified and subjected to sequencing reaction using ABI 3500 XL genetic analyser. The sequencing mix composition used was 4 μL of Big Dye terminator ready reaction mix, 1 μL of the template (100 ng/μL), 2 μL of primer (10 pmol/μL), and 3 μL of Milli-Q water. The PCR cycling parameters were 25 cycles of initial denaturation (96 °C for 1 min), denaturation (96 °C for 10 s), hybridization (50 °C for 5 s), elongation (60 °C for 4 min) using a POP-7™ polymers 50 cm capillary array [25]. The raw sequencing data analysis was done with Sequencing Analysis v 5.4 and imported into Chromas software for processing. The 16S rRNA sequence was used to carry out the BLAST alignment search tool of the National Center for Biotechnology Information (NCBI) Genbank database. Multiple sequence alignment was done using the ClustalW software tool and the Phylogenetic tree was constructed using http://ebi.ac.uk/.

### PCR amplification of pullulanase gene

The oligonucleotide primers used for the amplification of the pullulanase gene were forward 5’-ATGCCGGGTATCAGCCGCCC-3’ and reverse 5’- TCACCCTTTTGGTTCGTATAAAAC- 3’ using the isolated genomic DNA as a template. The PCR amplification was carried out as described by Zidani et al. [24] with the following cycling profile: initial denaturation step (94 °C, 5 min), 30 cycles of denaturation (94 °C, 60 s), annealing (50 °C, 90 s), initial extension step (72 °C, 150 s), and a final extension (72 °C, 20 min). For the analysis of DNA, agarose gel electrophoresis was carried out under standard conditions. The amplified gene was sequenced using ABI 3500 XL Genetic Analyzer.

### In silico analysis of pullulanase gene

The EXPASy Bioinformatics Resource Portal (https://web.expasy.org/translate/) was used to translate the nucleotide sequence of the pullulanase gene into its corresponding protein primary sequence, followed by identification of the best reading frame. A series of freely available online bioinformatics tools, including NCBI BLASTp (http://blast.ncbi.nlm.nih.gov/Blast) and CD tools [23, 26], were used to identify conserved domains and closely matched pullulanase sequences. Multiple sequence alignment and phylogenetic tree construction were done using EMBL-EBI Clustal W2 and phylogenetic tools available at http://www.ebi.ac.uk/Tools, and molecular evolutionary genetics analysis (MEGA 11) software, respectively [26, 27]. The 3D structure of the pullulanase was predicted using the homology Swiss modeling server (https://swissmodel.expasy.org/), while visualization was done using PYMOL tool.

Several validation tools were applied to ensure the excellence and durability of the PulA protein structure. The stereochemical quality of the structure was evaluated using Procheck [28–30], which assesses several geometric parameters. ProSa [31, 32] and Verify-3D [33, 34] scores were generated to assess the agreement between the 3D models and the relevant amino acid sequences. Furthermore, restriction mapping of the pullulanase gene sequence was carried out using the bioinformatics tool available online at (http://nc2.neb.com/NEBcutter2/). The molecular weight (MW) and isoelectric point (pI) of the deduced protein sequence were determined using the ExPASy—Compute pI/MW tool available online at http://web.expasy.org/computepi/. The aliphatic index of the deduced protein was determined using the ExPASy-ProtParam tool (https://web.expasy.org/protparam/) [35]. Additionally, the hydrophilic/hydrophobic characteristics and transmembrane segment predictions of the deduced protein sequence were carried out using in silico applications available at http://harrier.nagahama-i-bio.ac.jp/sosui/sosui_submit.html [36] and http://www.cbs.dtu.dk/services/TMHMM-2.0 [37], respectively. The thermodynamic properties of the protein were determined using scoop prediction as described by Pucci et al. [38].

## Results

### Isolation and identification of *B. licheniformis* Strain FAO.CP7

*B. licheniformis* Strain FAO.CP7 was isolated from a cocoa pod and characterized by its biochemical and morphological properties. We found that the strain FAO.CP7 was Gram-positive, rod-like, and spore-forming which is similar to that of *Bacillus spp.* The strain grows at optimal pH of 6.0 and a temperature of 50 °C. It is positive for gelatin hydrolysis, nitrate reduction, and catalase tests. It utilizes citrate and some sugars including glucose, lactose, sucrose, starch, maltose, and fructose but not arabinose or D-xylose. It is negative to urease reaction, methyl red (MR), and oxidase tests (Table 1).

**Table 1:** Biochemical, morphological, and physiological characteristics of strain FAO.CP7

Experiments	Results
Gram Reaction	Gram-Positive Rod
Spore	+
Optimal Temperature	50 ^0^C
Optimal pH	6.0
Catalase	+
Starch Hydrolysis	+
Indole Production	-
Urease Activity	-
Citrate Utilization Test (CU)	+
Gelatin Hydrolysis	+
Nitrate Reduction	+
Voges- Proskauer Test (VP)	+
Methyl Red Test (MR)	-
Oxidase Test	-
Utilization of D-Glucose	+
Utilization of D-Lactose	+
Utilization of D-Xylose	-
Utilization of Sucrose	+
Utilization of Starch	+
Arabinose	-
Maltose	+
Fructose	+

### Phylogenetic analysis

#### Phylogenetic analysis for *Bacillus licheniformis* FAO.CP7 strain

**Fig. 1:**
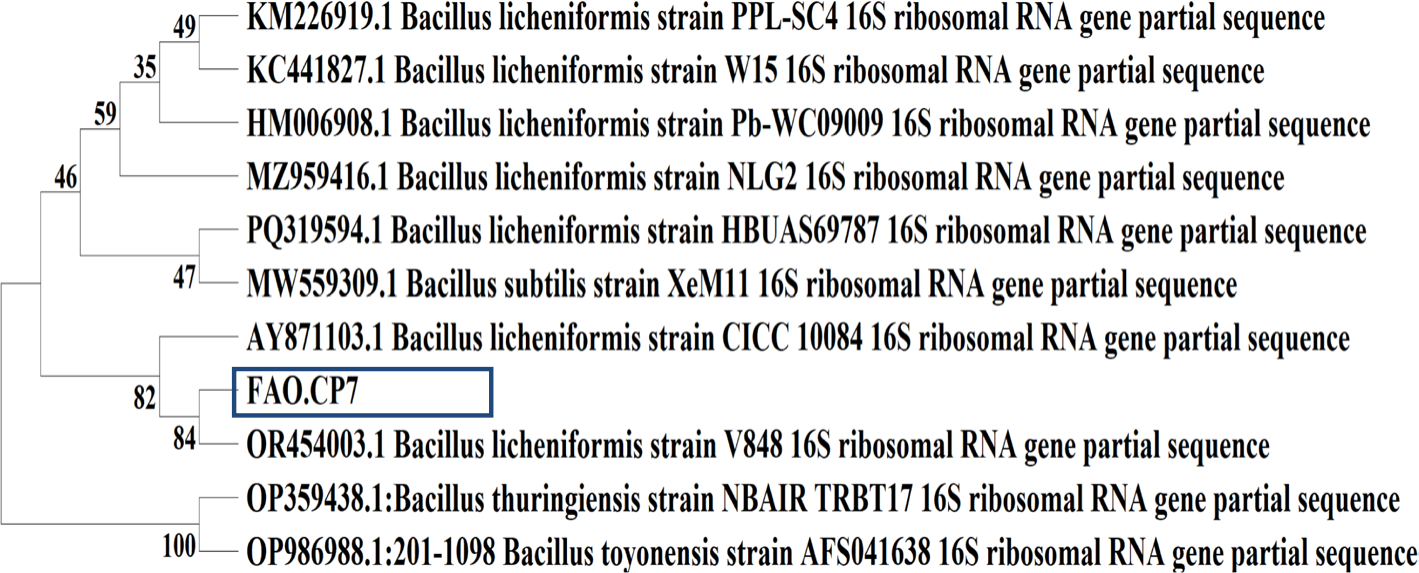
Phylogenetic tree of the nucleotide sequence of bacterial isolate FAO.CP7. The isolate has been deposited in the NCBI database with accession number MN150530.1

For further molecular identification of the FAO.CP7 strain, the 16S rRNA gene amplicons were subjected to 1% agarose gel electrophoresis to ascertain the presence of an amplified gene. The 16S rRNA gene sequence (760 base pairs) was deposited in GenBank (accession number MN150530.1) (Supplementary Fig. 1) and was analysed versus others in the GenBank sequence database. Alignment of the 16S rRNA gene of *B. licheniformis* strain FAO.CP7 with the 16S rRNA sequences from reference strains in GenBank was done using the NCBI BLASTn program. The BLAST result confirmed a very close similarity of the 16S rRNA gene sequence; with *B. licheniformis* strain FAO.CP7 shares at least 98% homology with the other *Bacillus spp.* (Table 2). The phylogenetic tree is based on the sequence of *B. licheniformis* strain FAO.CP7 and the sequences of the reference strains in the GenBank database are shown in Fig. 1. Analysis of our BLAST result in Table 2 showed that *B. licheniformis* Strain Pb-WC09009 (accession number HM006908.1), *B. licheniformis* Strain PPL-SC4 (accession number KM226919.1) *B. licheniformis* Strain CICC 1008416S (accession number AY871103.1) and *B. licheniformis* Strain W1516S (accession number KC441827.1) were 99% similar to FAO.CP7 strain. However, the phylogenetic tree in Fig. 1 revealed that *B. licheniformis* strains Pb-WC09009 and PPL-SC4 were the closest to strain FAO.CP7 while *B. licheniformis* strains CICC 1008416S and W1516S were relatively further away on the tree.

**Table 2:** Blast data of contig sequence of *B. licheniformis* strain FAO.CP7 shows the alignment view using a combination of NCBI GenBank

Matched Protein/Organism	Max Score	Total Score	Query Cover	E-Value	Identity	Accession Number	
*B. licheniformis* Strain Pb-WC09009 16S Ribosomal RNA Gene, Partial Sequence	1305	1305	99%	0.0	99%	HM006908.1	
*B. licheniformis* Strain PPL-SC4 16S Ribosomal RNA Gene, Partial Sequence	1305	1305	99%	0.0	99%	KM226919.1	
*B. licheniformis* Strain CICC 1008416S Ribosomal RNA Gene, Partial Sequence	1303	1303	99%	0.0	99%	AY871103.1	
*B. licheniformis* Strain W1516S Ribosomal RNA Gene, Partial Sequence	1301	1301	99%	0.0	99%	KC441827.1	
*Bacillus sp*. H15-1. Complete Genome	1297	10,384	99%	0.0	98%	CP018249.1	
*B. licheniformis* strain DSM 13 16S ribosomal RNA, partial sequence	1297	1297	99%	0.0	98.41%	NR118996.1	
*B. licheniformis* strain D12 16S ribosomal RNA gene, partial sequence	1297	1297	99%	0.0	98.41%	KU551144.1	
*B. licheniformis* Strain B13 16S Ribosomal RNA Gene, Partial Sequence	1297	1297	99%	0.0	98.41%	KC510197.1	
*B. licheniformis* strain BCRC 11702 16S ribosomal RNA, partial sequence	1297	1297	99%	0.0	98.41%	NR_116023.1	
*Bacillus subtilis* strain 13 16S ribosomal RNA gene, partial sequence	1292	1297	97%	0.0	98.66%	JF322927.1	
*B. licheniformis* WX-02 Genome	1292	10,391	99%	0.0	98%	CP012110.1	
*B. licheniformis* strain NCTC10341 genome assembly, chromosome	1293	10,299	99%	0.0	98.28%	LR134392.11	
*B. licheniformis* strain ATCC 9789 chromosome, complete genome	1293	10,325	99%	0.0	98.28%	CP023729.1	
*Bacillus sp. s*train P4(1) 16S ribosomal RNA gene, partial sequence	1293	1293	99%	0.0	98.28%	MF193496.1

#### Phylogenetic analysis for pullulanase from *Bacillus licheniformis* FAO.CP7 strain

**Fig. 2:**
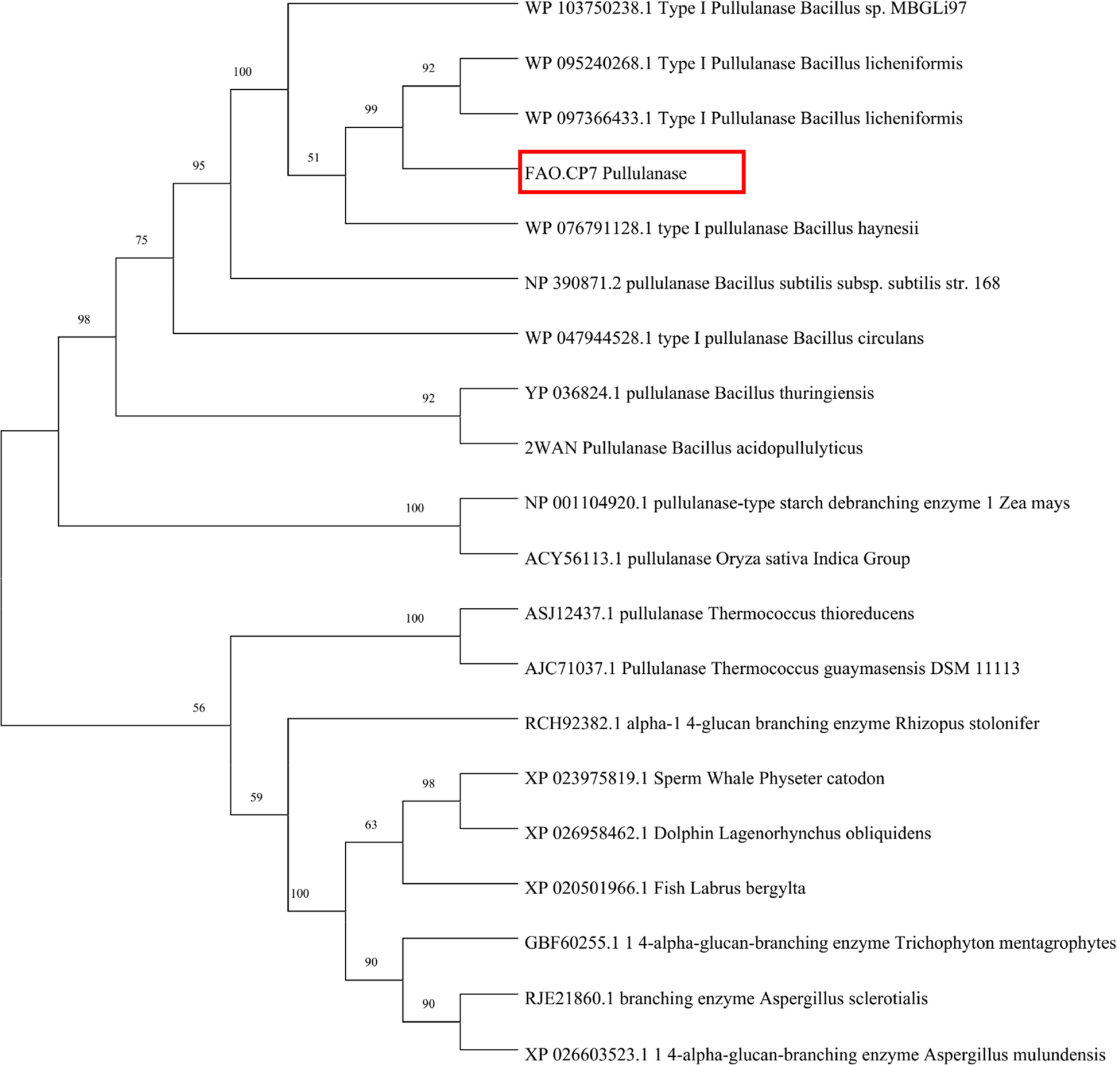
Evolutionary relationship between *B. licheniformis* FAO.CP7 pullulanase gene (*Pul A*) and those in plants, fungi, bacteria, lower animals, and insects in NCBI GenBank using Mega 11

To investigate the evolutionary relationship of *B. licheniformis* FAO.CP7 pullulanase gene (*Pul A*) with those in GenBank database, we amplified *Pul A* gene and deposited it on NCBI database with accession number PQ360904. Then, using the EXPASy Bioinformatics Resource Portal (https://web.expasy.org/translate/), we translated the *Pul A* (PQ360904) nucleotide sequence (2247 base-pairs) to 748 amino acid residues (Supplementary Fig. 2). Alignment of various pullulanases from GenBank and the phylogenetic analysis showed that the protein is more closely related to pullulanase of bacterial origin especially *B. licheniformis* than pullulanase of fungal, insect or animal origin (Fig. 2). The pairwise identities of *B. licheniformis* FAO.CP7 pullulanase protein sequence versus pullulanase protein sequences from the other *Bacillus spp.* ranged from 85 to 62% (Table 3). Our result revealed the gene has YNWGYNP, a signature motif which is peculiar to Type I pullulanases (Table 4) and also reveals the presence of some conserved regions in the aligned pullulanase (Supplementary Fig. 3). The amino acid residues present in the conserved domain include aspartate (D), histidine (H), arginine (R), and glutamate (E) amongst others (Table 4).

**Table 3:** BLASTp result of *B. licheniformis* FAO.CP7 PulA protein-protein matches and source organisms

**Matched Protein/Organism**	**Max Score**	**Total Score**	**Query Cover**	**E-Value**	**Percentage Identity**	**Accession Number**
Type I pullulanase [B. licheniformis]	1247	1247	100%	0	85.18%	WP_095240268.1
Type I pullulanase [B. licheniformis]	1246	1246	100%	0	85.05%	WP_097366433.1
MULTISPECIES: Type I pullulanase [Bacillus sp]	1246	1245	100%	0	85.05%	WP_151297381.1
Type I pullulanase [B. licheniformis]	1245	1245	100%	0	85.05%	WP_016885603.1
Type I pullulanase [B. licheniformis]	1245	1245	100%	0	84.67%	WP_107661658.1
Type I pullulanase [B. licheniformis]	1244	1244	100%	0	85.05%	WP_011198203.1
Type I pullulanase [B. licheniformis]	1243	1243	100%	0	85.05%	WP_075178235.1
Type I pullulanase [B. licheniformis]	1243	1243	100%	0	84.91%	WP_144564795.1
Type I pullulanase [Bacillus haynesii]	1179	1179	100%	0	80.51%	WP_043926817.1
Type I pullulanase [Bacillus paralicheniformis]	1160	1160	100%	0	79.44%	WP_023855523.1
Type I pullulanase [Bacillus swezeyi]	914	914	100%	0	62.22%	WP_148958029.1

**Table 4:** Regions conserved among type 1 Pullulanase of Bacillus Genus

Source	Accession Number	**YNWGYDP**		**REGION I**		**REGION II**		**REGION III**		**REGION IV**	
		Position	Sequence	Position	Sequence	Position	Sequence	Position	Sequence	Position	Sequence
*B.licheniformis* FAO.CP7	MN150530	323	YNWGYNP	376	**D**VVYN**H**T	445	GF**R**F**D**LM	477	G**E**GWD	556	YVSK**HD**N
*Bacillus sp*. CICIM 263	JX018171	461	YNWGYDP	505	**D**VVYN**H**	576	GF**R**F**D**LM	608	G**E**GWD	688	YAEA**HD**N
*Bacillus cereus*	YP_084052	425	YNWGYDP	468	**D**VVYN**H**	539	GF**R**F**D**LM	571	G**E**GWD	657	YVEA**HD**N
*Bacillus sp*. Strain KSM-1876	AB49812	1349	YNWGYNP	1497	**D**VVFN**H**T	1460	GF**R**F**D**MM	1492	G**E**GWV	1475	YIEA**HD**N
*Bacillus megaterium*	WP_060747396.1	569	FNWGYDP	616	**D**VVYN**HV**	638	GF**R**F**D**MN	715	G**E**GWDL	801	YVEA**HD**N
*B. stearothermophilus*	E03513	572	YNWGYNP	619	**D**VVYN**H**T	690	GF**R**F**D**LM	722	G**E**GWD	461	YVSK**HD**N
*Bacillus subtilis* 168	NC_000964.3	288	YNWGYNP	335	**D**VVFN**H**V	402	GF**R**F**D**LL	434	G**E**GWD	520	YVES**HD**N
*Bacillus thuringiensis*	EEM34934.1	427	YNWGYDP	474	**D**VVYN**H**M	541	GF**R**F**D**LM	573	G**E**GWD	659	YVEA**HD**N
*Bacillus circulans*	WP_047944528.1	292	YNWGYNP	339	**D**VVYN**H**V	406	GL**R**F**D**LM	438	G**E**GWD	524	YVES**HD**N
*B. acidopullulyticus*	2WAN_A	505	YNWGYDP	482	**D**VVYN**H**T	550	GF**R**F**D**LM	581	G**E**PWT	662	YVTS**HD**N
							*		*		*

The underlined amino acid residues are the highly conserved residues

While * stands for Catalytic triad (Asp-Glu-Asp)

### Structural modeling and bioinformatic analysis

**Fig. 3:**
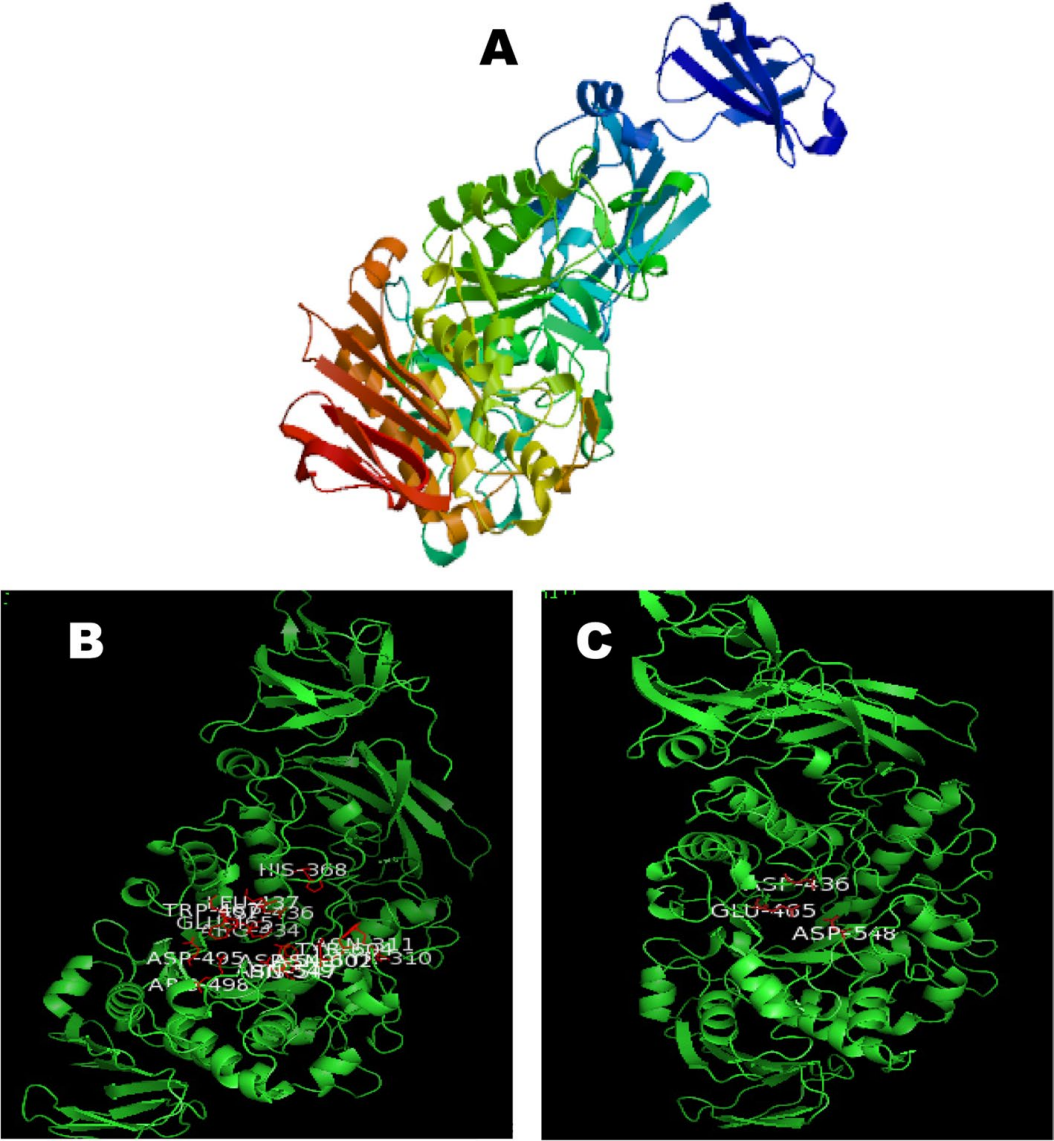
Structural analysis of Pullulanase. **A** The predicted 3D-structure modelling of pullulanase using 3D Swiss modelling program based on the pullulanase from *Bacillus subtilis* Str. *168* (PDB: 2E9B) while visualization was done using PYMOL (**B**) Structure indicating the amino acid residues at the active site of Pullulanase (which are Tyr^310^, Asn^311^, His^363^, Arg^434^, Asp^436^, Leu^437^, Glu^465^, Trp^467^, Asp^495^, Arg^498^, His^547^, Asp^548^, Asn^549^, Asn^602^ and Tyr^604^) and (**C**) The amino acids at the catalytic triad of Pullulanase (namely Asp^436^, Glu^465^, Asp.^548^)

The 3D structure of *B. licheniformis* FAO.CP7 pullulanase (*PulA*) was modeled using Swiss-Model (https://swissmodel.expasy.org/) (Fig. 3A). Structural analysis identified 15 active-site residues (Tyr310, Asn311, His363, Arg434, Asp436, Leu437, Glu465, Trp467, Asp495, Arg498, His547, Asp548, Asn549, Asn602, and Tyr604), with Asp436, Glu465, and Asp548 forming the catalytic triad (Fig. 3B-C).

**Fig. 4:**
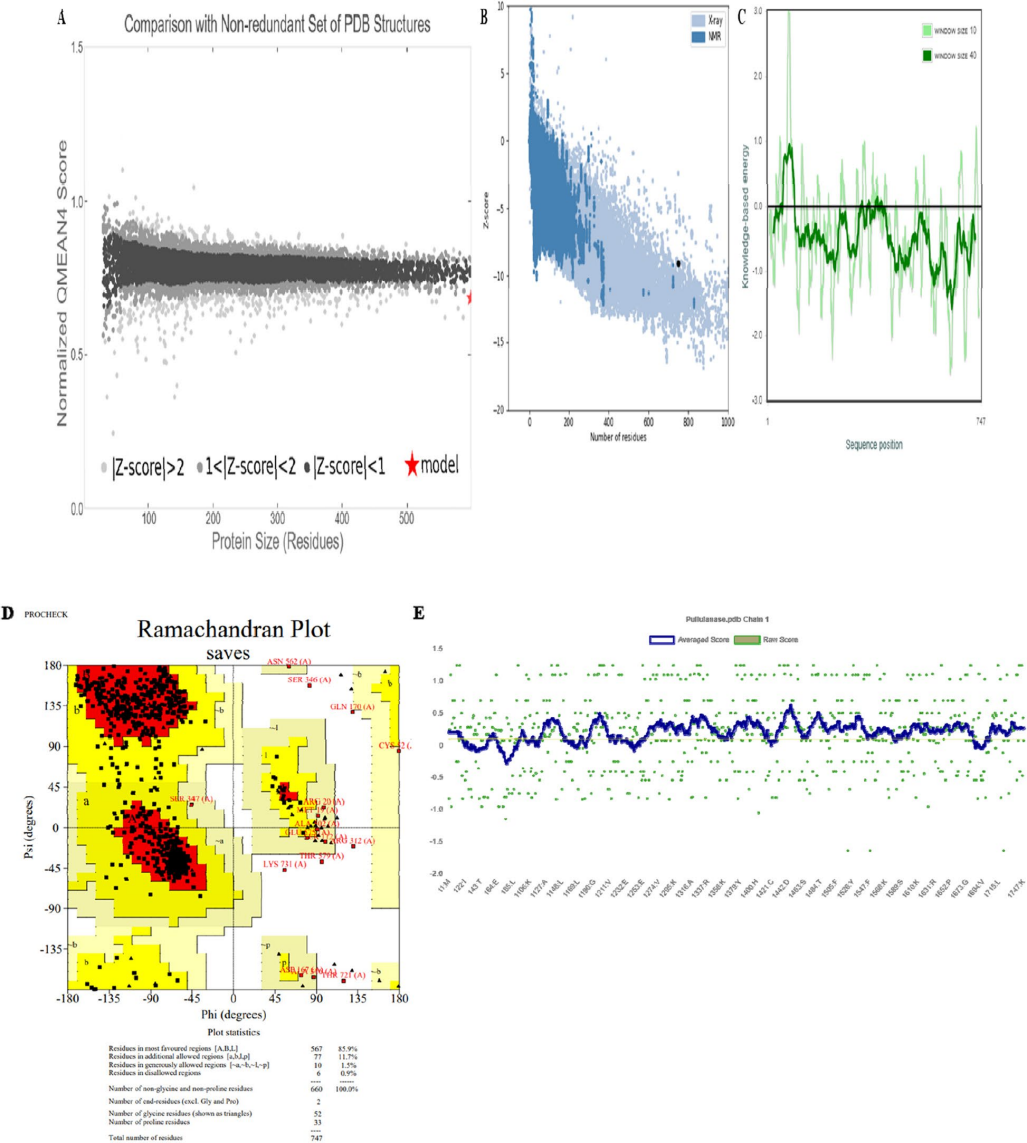
Validation results of the PulA protein model. **A** QMEAN4 score: -2.25. QMEAN4 is a linear combination of four statistical potential terms, trained to predict global lDDT scores in the range [0, 1]. The displayed value is a Z-score transformation of the original QMEAN4 score, providing a comparison with values typically observed for high-resolution X-ray structures. **B** ProSA-web Z-scores for all protein chains in the PDB, determined by X-ray crystallography (light blue) or NMR spectroscopy (dark blue), and plotted against chain length. The plot includes only chains with fewer than 1000 residues and a Z-score ≤ 10. The Z-score for PulA is highlighted as a large black dot. **C** Energy plot of PulA: Residue energies averaged over a sliding window (window size = 40, default) are plotted as a function of the central residue in the window, reflecting the large size of the protein chain. **D** Procheck analysis of PulA: 85.9% of residues fall within the favored region, indicating a high degree of stereochemical accuracy. **E** Verify3D analysis of PulA: 77.38% of residues have an averaged 3D-1D score ≥ 0.1, passing the quality threshold

To assess the quality and reliability of the homology model, we performed structural validation using multiple bioinformatics tools. The QMEAN score (-2.25), computed via Swiss-Model, indicates a model quality within the acceptable range for homologous proteins [39]. Further global and local model validation using ProSA-web yielded a Z-score of -9.03, suggesting that the predicted structure aligns well with experimentally determined protein structures (Fig. 4A-C). To further validate the model’s stereochemical accuracy, the Ramachandran plot was generated using Procheck. The plot revealed that 85.9% of the residues were in the most favored regions (A, B, L), 11.7% in additional allowed regions (a, b, l, p), 1.5% in generously allowed regions (~ a, ~ b, ~ l, ~ p), and only 0.9% in disallowed regions. These statistics align with expected quality thresholds, as models with over 90% of residues in favoured regions are considered of high quality (Fig. 4D). To evaluate the degree of correspondence between the respective amino acid sequences and the 3D models, VERIFY3D scores were generated (Fig. 4E). The analysis revealed that 77.38% of residues had an averaged 3D-1D score ≥ 0.1. This result, while slightly below the optimal threshold of 80%, suggests that the predictions generated by the model may not fully correspond with the characteristics of a well-folded protein, particularly in some regions. However, the majority of residues still meet the required standards, supporting the overall reliability of the model despite the minor discrepancy.

**Fig. 5:**
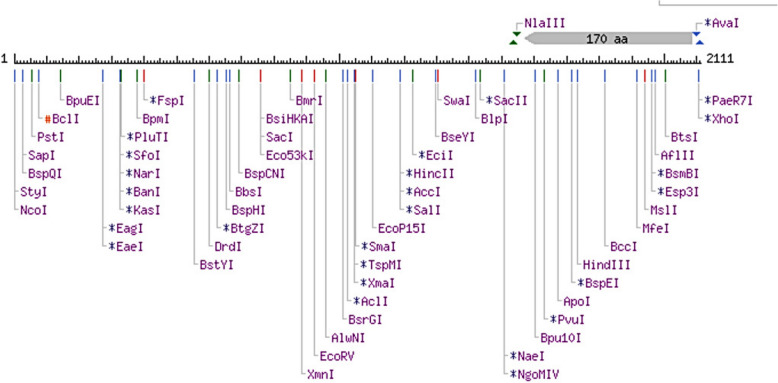
Endonuclease restriction map of the *B. licheniformis* FAO.CP7 pullulanase gene (*Pul A*)

**Fig. 6:**
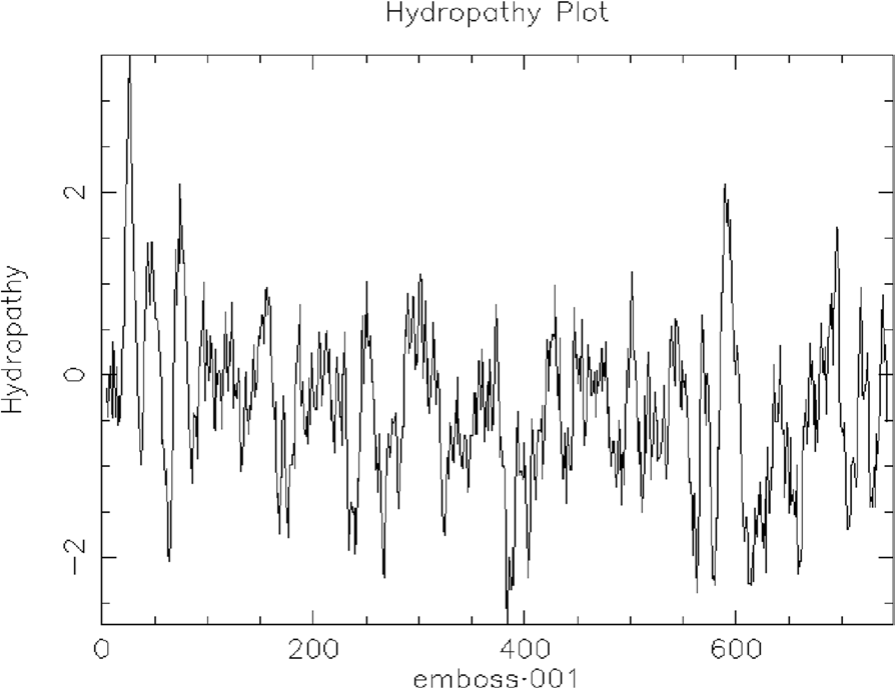
Hydrophobicity prediction of *B. licheniformis* FAO.CP7 pullulanase. The average hydrophobicity is -0.37 (below zero)

**Fig. 7:**
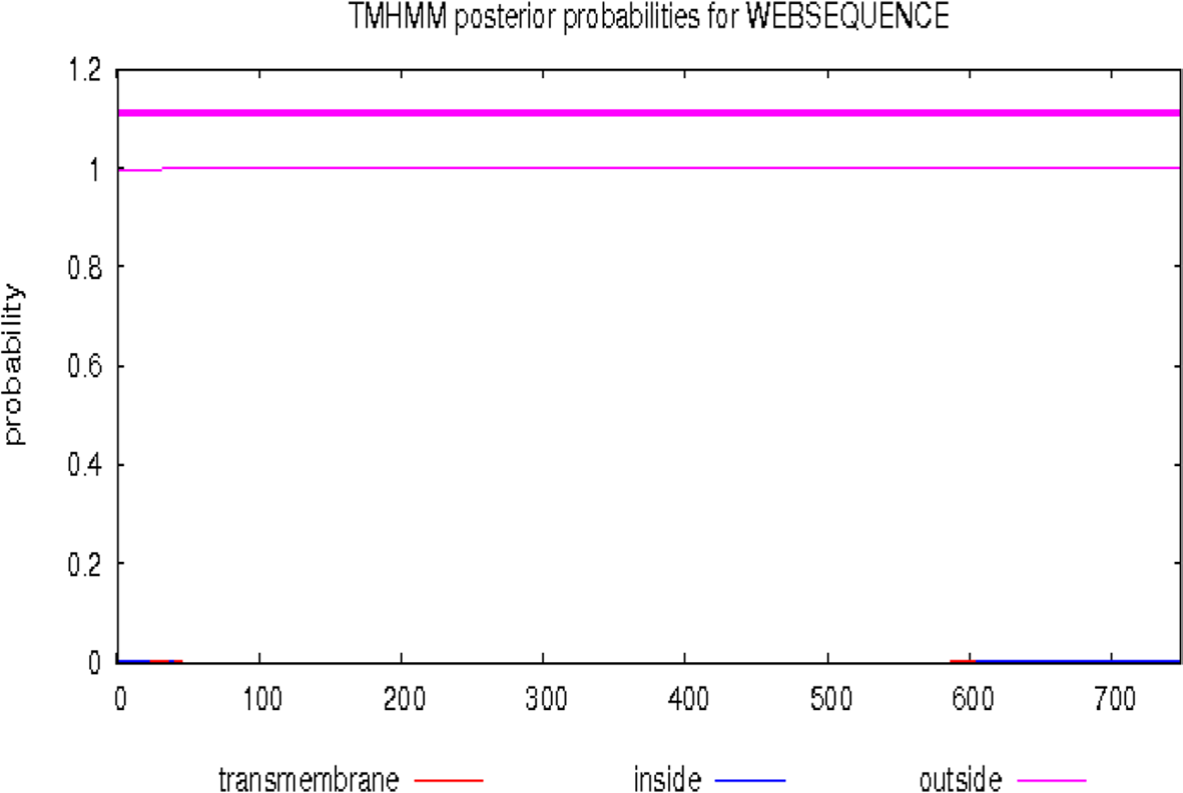
In silico analysis of the transmembrane segments in pullulanase (*Pul* A). No transmembrane helix was found in the pullulanase protein which indicated that the protein is a non-transmembrane protein but rather a soluble extracellular protein

**Fig. 8:**
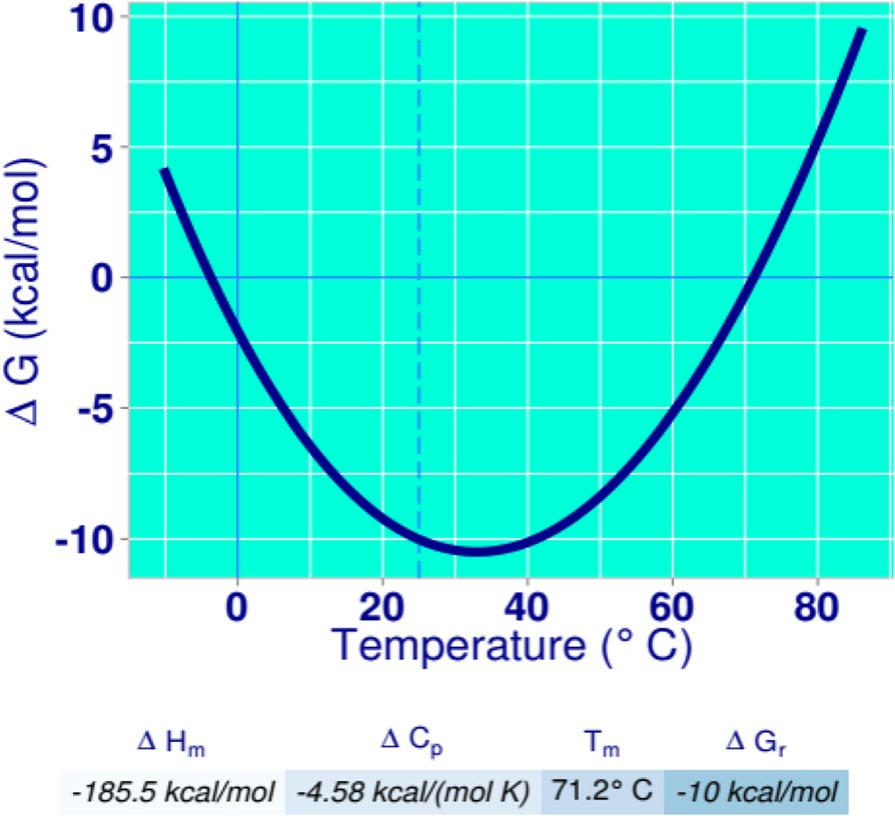
Thermodynamic properties of Pullulanase (*Pul* A) from *B. licheniformis* FAO.CP7 strain using Scoop prediction package

Further physicochemical analysis of *PulA* revealed a molecular weight (MW) of 82.39 kDa and an isoelectric point (pI) of 6.47 (Table 5). As shown by the endonuclease restriction linear map in Fig. 5, a wide array of restriction enzymes (such as HindIII, PstI, EagI, SacI, EcoRV, SalI, SmaI, and BcLI among others) can be used to cut the Pul A gene, thus suggesting that PulA can easily be manipulated during cloning and gene expression experiments. However, there are some exceptions, as restriction enzymes like EcoRI, BamHI, NotI, Xbal, SpeI, NheI, and KpnI will not be able to cut the *PulA* gene (Fig. 5). The hydrophobicity score (-0.37), determined via SOSUI (http://harrier.nagahama-i-bio.ac.jp/sosui/sosui_submit.html), suggests that *PulA* is a soluble enzyme (Fig. 6), consistent with its non-transmembrane nature (Fig. 7). Additionally, thermodynamic analysis using SCooP predicted a melting temperature (Tm) of 71.2 °C, a ΔCp of -4.58 kcal/(mol·K), and an enthalpy change (ΔHm) of -185.5 kcal/mol (Fig. 8). The aliphatic index (77.06) further supports PulA’s thermostability, as values within 42.08–90.68 indicate proteins capable of maintaining structural integrity under elevated temperatures [40]. Together, these findings confirm that the homology model is reliable and that *PulA* is a soluble, thermostable enzyme with potential for biotechnological applications.

**Table 5:** Isoelectric point and molecular weight of *B. licheniformis* FAO.CP7 pullulanase gene (*Pul A*)

Pullulanase	Number of the nucleotide sequence	Number of amino acid residues	Molecular weight (Dalton)	Monoisotopic mass	Isoelectric point (pI)
*B. licheniformis* FAO.CP7 pullulanase (*Pul* A)	2247	748	82,347.48	82,394.25	6.47

## Discussion

Pullulanase (EC 3.2.1.41) play a critical role in starch-based industries by enhancing the release of abundant energy present in branched polysaccharides during the saccharification process [3]. Pullulanases have been derived and characterized from different microbial sources [8–15]. However, the diversity and properties of pullulanase in *B. licheniformis* which is associated with cocoa pod waste have not been analysed earlier. Thus, in the present study, *B. licheniformis* strain FAO.CP7 (accession No: MN150530.1.) was isolated and molecularly characterized. Also, the phylogenetic, structural, and functional analysis of pullulanase (*PulA*) from *B. licheniformis* strain FAO.CP7 was carried out using several bioinformatics tools.

The pullulanase-producing *B. licheniformis* strain FAO.CP7 which was isolated from cocoa pod wastes is Gram-positive. It was coccus and rod-like under the microscope, and it formed smooth and bright yellow colonies on a nutrient medium. The optimal temperature and pH for growth of *B. licheniformis* strain FAO.CP7 were 50 °C and 6.0, respectively. The microbe tested positive for catalase, nitrate reduction, gelatin hydrolysis, and Vogus-Proskauer (VP) test. Meanwhile, Methyl-Red (MR) test and indole production were negative. The strain can utilize the following substrates as carbon sources: D-glucose, D-lactose, D-maltose, sucrose, and starch but not D-xylose and arabinose (see Table 1).

The identification of the strain of interest can be achieved by both conventional and molecular methods. In this study, both methods were used to identify the bacterial isolate and the consensus sequence obtained were then compared with the NCBI gene bank database using the BLAST search program (http;//www.ncbi.nlm.nih.gov) [41, 42]. The BLAST analysis of 16S rRNA sequences showed that *B. licheniformis* strain FAO.CP7 has a close relationship with *B. licheniformis* Pb-WC09009 (99%), *B. licheniformis* PPL-SC4 (99%), *B. licheniformis* CICC 10084 (99%), *B. licheniformis* W15 (99%) (see Table 2). This observation is consistent with a previous study [19]. Furthermore, the sequenced 16S rRNA region of *B. licheniformis* strain FAO.CP7 was used for the construction of the phylogenetic tree to know the genetic relatedness and evolutionary origin between the bacterial isolate and the closely related homologs of identified bacteria. The grouping in the phylogenetic tree showed that strains having similar sequences were clustered in the same group and as a result, they are considered as close relatives [43, 44]. Based on the physiological, biochemical, and 16S rRNA alignment analyses, the strain was identified to be a member of the *B. licheniformis* family and deposited as *B. licheniformis* FAO.CP7 (accession No: MN150530.1).

Herein, we amplified the pullulanase (*Pul A*) gene from the genome of the isolated *B. licheniformis* FAO.CP7 strain, and analyzed its amino acid sequence. Our data revealed that the *Pul A* gene consists of 748 amino acid residues. The deduced amino acid sequence of the *PulA* protein showed the highest similarity with type I pullulanase from *B. licheniformis* WP_095240268.1 (85.18%) (Table 3). Phylogenetic analysis of the *Pul A* gene amplified from *B. licheniformis* FAO.CP7 showed that it is more closely related to pullulanase genes from bacteria than from fungi, insects, or animals. As with many enzymes from the α-amylase GH13 family, *B. licheniformis* FAO.CP7 *PulA* contains conserved residues in its substrate binding sites, typical of thermophilic strains of *Bacillus* (Table 4, Fig. 3) [45, 46]. The catalytic region of pullulanase enzymes generally comprises three amino acid residues—two aspartates and one glutamate—which are involved in hydrolyzing α-1, 6-glycosidic linkages [47–50]. Our results confirm this pattern, identifying Asp436, Glu465, and Asp548 as key catalytic residues in *B. licheniformis* FAO.CP7 *PulA* (Fig. 3), a finding consistent with previous studies on related thermostable pullulanase proteins from *Anoxybacillus* species [51, 52]. Also, the conserved region identified in this study, which includes the amino acid sequence YNWGYNP, is similar to regions found in type I pullulanases [48, 53–56]. This region is known to play a role in substrate binding and catalytic activity [49], specifically by cleaving the α-1,6 glycosidic bonds of pullulan [49, 55, 57]

Further characterization through in silico analysis revealed key physicochemical properties of the *B. licheniformis* FAO.CP7 pullulanase. The molecular weight and isoelectric point were determined to be 82.39 kDa and 6.47, respectively (Table 5). These values are consistent with previous studies, such as that of Kashiwabara et al. [58] and Al-Mamoori et al. [55], and suggest that the enzyme is most active in neutral to mildly acidic environments, similar to pullulanases from other sources such as *Exiguobacterium acetylicum, Sulfolobus acidocaldarius, Paenibacillus barengoltzii*, white edible mushrooms, *Geobacillus thermocatenulatus, Geobacillus thermopakistaniensis* [59–63]. Additionally, the hydrophobicity score of -0.37 indicates that the protein is soluble, further confirming that *Pul A* is a non-transmembrane, extracellular enzyme, in agreement with the findings of Hang et al. [64] and Meng et al. [65].

The structural validation of *Pul A* confirms its reliability for industrial applications, with QMEAN and ProSA-web analyses indicating strong structural integrity, while the Ramachandran plot supports its well-folded conformation. However, minor deviations in VERIFY3D suggest potential flexibility in certain regions, which could influence enzyme stability and catalytic efficiency under industrial conditions. Similar observations in bacterial lipases and GH11 xylanases highlight the impact of subtle structural variations on enzyme performance, emphasizing the need for precise modeling [66, 67]. Given *PulA*’s critical role in hydrolyzing α-1, 6-glycosidic linkages, ensuring structural robustness is essential for optimizing its function in biotechnological applications such as starch processing and biofuel production [68]. Recent advances in enzyme engineering demonstrate that targeted modifications informed by molecular dynamics simulations can enhance thermostability and activity [69], suggesting that further computational refinements could improve *Pul* A’s industrial potential.

The thermostability of *B. licheniformis* FAO.CP7 *Pul* A was further confirmed by a predicted melting temperature (Tm) of 71.2 °C (Fig. 7), coupled with an aliphatic index of 77.06. These properties suggest that *Pul A* is highly stable under elevated temperatures, making it an ideal candidate for high-temperature biotechnological applications. Notably, while *B. licheniformis* FAO.CP7 is classified as a mesophilic bacterium, its *Pul A* protein exhibits significant thermostability, with a Tm comparable to thermostable pullulanases from *Thermomonospora fusca* [69], *Bacillus stearothermophilus* [46, 70, 71], *Streptococcus thermophilus* [72], and *Fervidobacterium pennavorans*Ven5 [47, 71], *Zea mays* [73] and *Pyrococcus yayanosii* CH1 [74]. The high aliphatic index further supports *Pul* A’s stability at high temperatures.

In addition to its thermostability and solubility, *Pul A* is amenable to genetic modification, as indicated by the presence of multiple restriction enzyme recognition sites (HindIII, PstI, EagI, and SacI, etc.) that facilitate cloning and mutagenesis (Fig. 5). This genetic flexibility allows for the optimization of *Pul* A’s catalytic efficiency, substrate specificity, and thermal stability through protein engineering strategies, such as site-directed mutagenesis and directed evolution. These capabilities open avenues for tailoring *Pul A* for specific industrial applications, further enhancing its utility as a biocatalyst in high-temperature processes such as starch hydrolysis and biofuel production.

Overall, *B. licheniformis* FAO.CP7, isolated from cocoa pod waste, is a novel pullulanase-producing strain, identified through morphological, biochemical, and molecular analyses. The *Pul A* enzyme encoded by this strain shares characteristics with type I pullulanases and exhibits promising thermostability and solubility. These features, combined with its genetic flexibility, position *Pul*A as a potential candidate for industrial applications, including starch degradation and biofuel production. However, further in vitro studies are needed to confirm the molecular weight, thermostability, and melting temperature of *Pul* A, which will provide additional insights into its practical applications.

## Supplementary Information

Supplementary Material 1.

## Data Availability

16S rRNA gene has been deposited in NCBI/ GenBank database with accession number MN150530.1 and the pullulanase gene was deposited in NCBI database with accession number PQ360904.

## Abbreviations

GH13: Glycoside hydrolase 13
CPH: Cocoa pod husk
PulA: Pullulanase
VP: Vogus-Proskauer
T_m_: Melting temperature
