# Supplementary material for: In silico and structural analysis of Bacillus licheniformis FAO.CP7 pullulanase isolated from cocoa (Theobroma cacao L.) pod waste
Source: BMC Microbiol. 2025 Apr 30;25:261. doi: 10.1186/s12866-025-03958-w (PMC12042331; doi:10.1186/s12866-025-03958-w)
Supplement: Supplementary file 1 — Supplementary Material 1. [file 12866_2025_3958_MOESM1_ESM.doc]

**2.1 Identification and Characterization of Bacterial Isolate**

The starch-utilizing *Bacillus spp* isolated was identified using their cultural and morphological characteristics in different media. A distinct colony of isolates that grew on the culture plate was observed for their cultural and morphological features. This was then followed by microscopic examination of the bacterial isolate on the glass slide placed under the microscope and it was viewed under oil immersion to observe the gram reaction and shape. The cultural features examined included shape elevation, surface edge and consistency. Biochemical tests were employed to confirm the identification of the isolate [2].

**2.2               Biochemical Characterization of Isolates**

A 24-hour-old culture of each isolate was used for the biochemical test using conventional microbiological methods.

**2.2.1 Catalase Test**

         The principle of this is based on the possession of the enzyme catalase by some organisms which break down hydrogen peroxide, releasing oxygen in the form of gas bubbles. A sterile inoculating loop was used to pick a small portion of the colony under test and placed in a drop of 3 % hydrogen peroxide solution on a clean, free slide. A positive catalase reaction shows effervescence [1].

**2.2.2            Indole Test**

This was used to test the ability of the organism to degrade tryptophan into products, including indole pyruvic acid and ammonia. The test organisms were inoculated inside Bijou bottles containing 3 ml of sterile tryptone water. The organisms will be incubated at 35 - 37 ^0^C for 48 hours. This will be followed by the addition of 0.5 ml of Kovac’s reagent. A red colour on the surface layer was observed within 10 minutes, which indicated a positive test for indole [3]

**2.2.3 Citrate Utilization Test**

The test is based on the ability of an organism to use citrate as its only source of carbon. Simmon’s citrate agar was prepared in slop on Bijou bottles as recommended by the manufacturer (it can be stored at 2-8 ^0^C). Using a sterile straight wire, the sloop was first streaked with saline suspension of the test organism and then the butt stabbed. Incubated at 35 ^0^C for 48 hours and was observed in the medium for a bright blue colour. Bright blue indicates that the citrate is positive, but no change in the medium's colour means the citrate test is negative [2].

**2.2.4 Acid Production from Carbohydrates (Sugar Test)**

         This test detects the ability of an organism to ferment certain carbohydrates either in the presence or absence of oxygen. The sugars used included glucose, lactose, xylose, sucrose, starch, arabinose, maltose and fructose. A 9 ml portion of basal medium was pipetted in sterile McCartney bottles with 1 ml of sugar solution. 3 drops of phenol red were added, Durham tubes were inverted and placed into the mixture and sterilized in an autoclave at 121 ^0^C for 15 minutes, and each bottle was labelled and inoculated with the test organisms and incubated at 37 ^0^C for 24 – 48 hours. A bottle not inoculated was used as a control. The fermented sugar solution gave a yellow colouration, indicating a positive reaction, while the unfermented sugar solution retained its original colour, indicating a negative reaction.  Acid production in some cases was accompanied by carbon-dioxide evolution, which was visible in the Durham tube [6, 7].

**2.2.5 Oxidase Test**

         A piece of filter paper was soaked with a few drops of oxidase reagent. A colony of the test organism was smeared on the filter paper using the inoculating loop. If the organism is oxidase producing, the reagent will be oxidised to a deep purple colour [1].

**2.2.6.           Methyl Red Test**

Five ml of glucose phosphate broth (1 g glucose, 0.5 % KH_2_PO_4_, 0.5 % peptone and 100 ml distill H_2_O) was dispensed in clean test tubes and sterilized at 121 ^0^C for 15 minutes. The tube was inoculated with the test organism and incubated at 37 ^0^C for 48 hours. At the end of incubation, five drops of methyl red solution were added to each test tube. The appearance of red colour indicates a positive reaction [2].

**2.2. 7           Urease Test**

         Three ml (3 ml) sterile Christensen’s (modified) urea broth was prepared in a Bijou bottle with the aid of a sterilized inoculating loop; the Bijou bottle was heavily inoculated with the test organism. The preparation was incubated at 37 ^0^C for 3 days. A pink colouration indicates a positive urease test due to breakdown of urea by enzymes to give ammonia and carbon dioxide; the release of ammonia makes the medium alkaline as shown by change in colour of the indicator pink-red [1].

**2.2. 8           Nitrate Reduction Test**

The ability of bacteria to reduce nitrate to nitrites or further compounds was tested by inoculating respective bacteria isolates into different nitrate media/broth and they were incubated for 35 ^o^ C for 5-8 days, 5 drops of nitrate reagent A (sulphanilic acid reagent) and B (α – napthylamine) was added into the tubes respectively. When the solution turns red within 5 minutes of addition, it is positive. If the reagent did not turn red, then the addition of reagent C, zinc powder, is needed to further confirm the presence of nitrate or other compounds. If, after adding zinc powder, the organism did not reduce the nitrate in the broth, but the colour remains, that means the nitrate has been reduced to other Nitrogen compounds such as Nitrogen and ammonia [5].

**2.2. 9           Starch Hydrolysis**

Starch agar plates were inoculated with different bacterial isolates and incubated at 35 ^o^C for 2-3 days. After incubation, each plate was flooded with aqueous iodine and left for 30 seconds. A clear zone surrounding the colonies indicated a positive test, while a blue-black coloration indicated the presence of starch meaning the latter has not be hydrolyzed [4].

**2.2.10  Voges-Proskaeur test**

         This test detects the production of acetyl-methyl carbinol, which is the intermediate compound formed during the conversion of pyruvic acid to 2 3 butylene glycol. The organisms were grown in glucose phosphate medium as described for methyl red. One ml of the broth culture, 0.5 ml of 6 % naphthol was added, followed by 0.5 ml of 40 % KOH. A strong red colouration formed within 30 minutes indicates a positive reaction [2, 4].

Supplementary figure 1: Contig sequence of 16S rRNA gene of bacterial isolate FAO.CP7 with accession number MN150530.1.


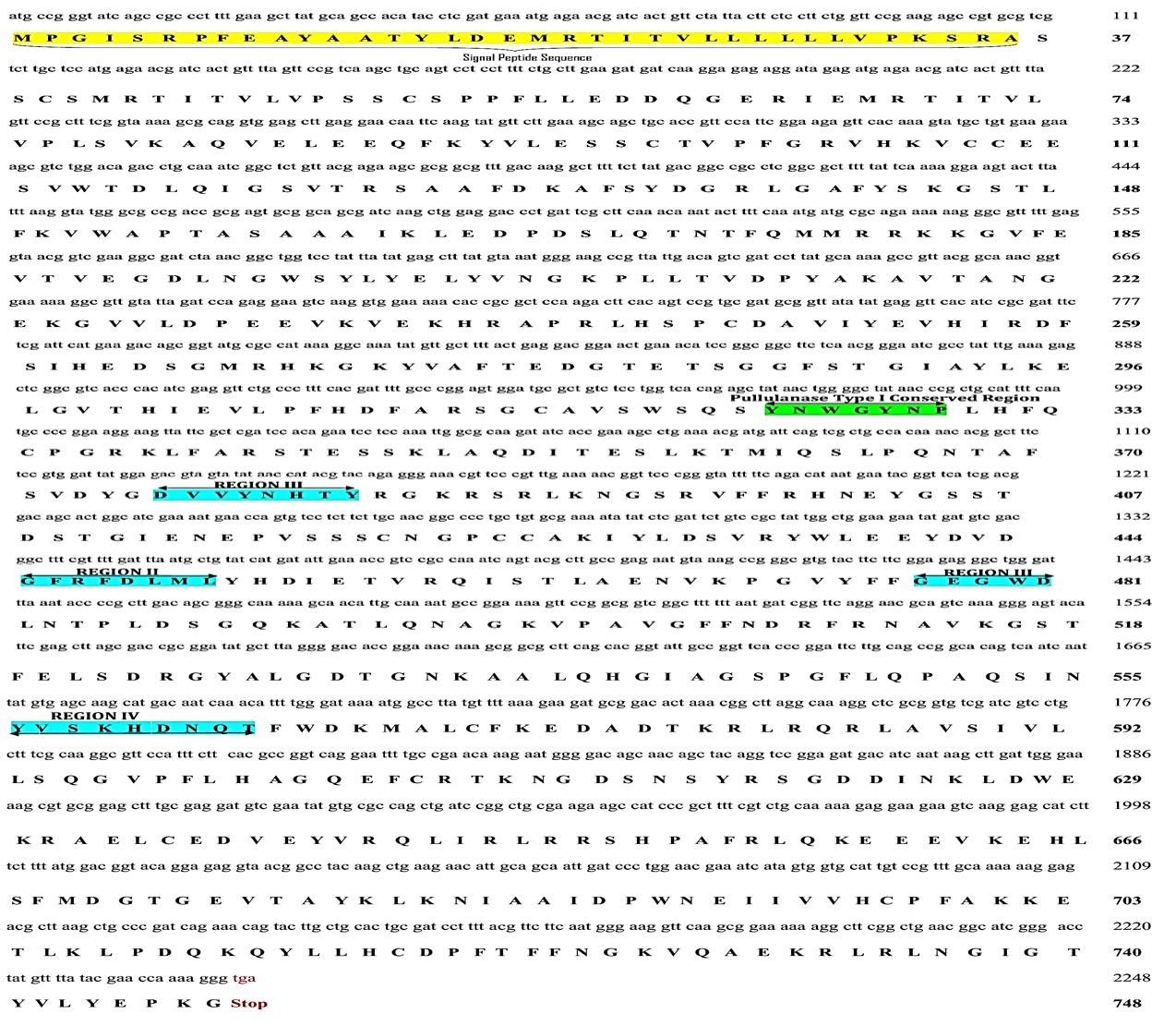


Supplementary figure 2

Supplementary figure 2. Deduced amino acid sequence of the *B. licheniformis* FAO.CP7 pullulanase gene (**PulA**) (PQ360904) using <https://web.expasy.org/translate/>. The yellow highlighted region represents the signal peptide region of the protein.

Pullulanase.FAO.CP7 ---------------------------------------------LSVKAQVELEEQFKY 15

WP_103750238.1 MPGISRPFEAYLDEMRTITVLVPKSRASSCSPPFLLEDDQGGKIELSVKATEELEEKFKY 60

WP_048350558.1 MPGISRPFEAYLDEMRTITVLVPKSRASSCSPPFLLEDDQGGKIELSVKATEELEEKFKY 60

WP_076791128.1 MPGISRPFEAYLDEMRTITVLVPKSHASSCTPPFFLEDDQGERIEISVKATEELEEQFKY 60

WP_009329394.1 MPGISRPFEAYLDEMRTITVLVPKSRASSCSPPFLLEDDQGERIELSVKAQVELEEQFKY 60

WP_095240268.1 MPGISRPFEAYLDEMRTITVLVPKSRASSCSPPFLLEDDQGERIELSVKAQVELEEQFKY 60

WP_097366433.1 MPGISRPFEAYLDEMRTITVLVPKSRASSCSPPFLLEDDQGERIELSVKAQVELEEQFKY 60

WP_101582708.1 MPGISRPFEAYLDEMRTITVLVPKSRASSCSPPFLLEDDQGERIELSVKAQVELEEQFKY 60

WP_107661658.1 MPGISRPFEAYLDEMRTITVLVPKSRASSCSPPFLLEDDQGERIELSVKAQVELEEQFKY 60

WP_016885603.1 MPGISRPFEAYLDEMRTITVLVPKSRASSCSPPFLLEDDQGERIELSVKAQVELEEQFKY 60

:**** ****:***

Pullulanase.FAO.CP7 VLESSCTVPFGRVHKVCCEESVWTDLQIGSVTRSAAFDKAFSYDGRLGAFYSKGSTLFKV 75

WP_103750238.1 VLESSRPVPFGRVHKVCCEESVWTDLQIGSVTRSAAFDKAFFYDGRLGAFYSKERTLFKV 120

WP_048350558.1 VLESSRPVPFGRVHKVCCEESVWTDLQIGSVTRSAAFDKAFFYDGRLGAFYSKECTLFKV 120

WP_076791128.1 ILESSRTVPFGRVHKVCCEESVWTDLQIGSVTRSAAFDKAFFYDGRLGAFYSKERTLFKV 120

WP_009329394.1 VLESSCNVPFGRVHKVCCEESVWTDLQIGSVTRSAAFDKAFFYDGRLGAFYSKGSTLFKV 120

WP_095240268.1 VLESSCTVPFGRVHKVCCEESVWTDLQIGSVTRSAAFDKAFFYDGRLGAFYSKGSTLFKV 120

WP_097366433.1 VLESSCTVPFGRVHKVCCEESVWTDLQIGSVTRSAAFDKAFFYDGRLGAFYSKGSTLFKV 120

WP_101582708.1 VLESSCTVPFGRVHKVCCEESVWTDLQIGSVTRSAAFDKAFFYDGRLGAFYSKGSTLFKV 120

WP_107661658.1 VLESSCTVPFGRVHKVCCEESVWTDLQIGSVTRSAAFDKAFFYDGRLGAFYSKGSTLFKV 120

WP_016885603.1 VLESSCTVPFGRVHKVCCEESVWTDLQIGSVTRSAAFDKAFFYDGRLGAFYSKGSTLFKV 120

:**** ********************************** *********** *****

Pullulanase.FAO.CP7 WAPTASAAAIKLEDPDSLQTNTFQMMRRKKGVFEVTVEGDLNGWSYLYELYVNGKPLLTV 135

WP_103750238.1 WAPTASTAAIKLESPDSLQTNTFQMMRREKGVFEVTIEGDLNGWSYLYKLYVNGTPLLTV 180

WP_048350558.1 WAPTASTAAIKLESPDSLQTNTFQMMRREKGVFEVTVEGDLNGWSYLYKLYVNGTPLFTV 180

WP_076791128.1 WAPTASTAAIKLESPDSLQTNTFQMMRRKKGVFEVTVEGDLNGWSYLYELYVNGTPLLTV 180

WP_009329394.1 WAPTASAATIKLENPDSLQTNTFQMMRRKKGVFEVTVEGDLNGWSYLYELYVNGKPLLTV 180

WP_095240268.1 WAPTASAAAIKLEDPDSLQTNTFQMMRRKKGVFEVTVEGDLNGWSYLYELYVNGKPLLTV 180

WP_097366433.1 WAPTASAAAIKLEDPDSLQTNTFQMMRRKKGVFEVTVEGDLNGWSYLYELYVNGKPLLTV 180

WP_101582708.1 WAPTASAAAIKLEDPDSHQTNTFQMMRRKKGVFEVTVEGDLNGWSYLYELYVNGKPLLTV 180

WP_107661658.1 WAPTASAAAIKLEDPDSLQTNTFQMMRRKKGVFEVTVEGDLNGWSYLYELYVNGKPLLTV 180

WP_016885603.1 WAPTASAAAIKLEDPDSLQTNTFQMMRRKKGVFEVTVEGDLNGWSYLYELYVNGKPLLTV 180

******:*:****.*** **********:*******:***********:*****.**:**

Pullulanase.FAO.CP7 DPYAKAVTANGEKGVVLDPEEVKVEKHRAPRLHSPCDAVIYEVHIRDFSIHEDSGMRHKG 195

WP_103750238.1 DPYAKAVTVNGEKGVVIDPEEVKVEKYRAPSLHSPCDAVIYEVHIRDFSIHEDSGMRHKG 240

WP_048350558.1 DPYAKAVTVNGEKGVVIDPEEVKVKKYRAPSLHSPCDAIIYEVHIRDFSIHEDSGMRHKG 240

WP_076791128.1 DPYAKAVTANGEKGVVLDPDEVKVEKHRAPSLHSPCDAVIYEVHIRDFSIHEDSGMRHKG 240

WP_009329394.1 DPYAKAVTANGEKGVVLDPEEVKVEKHRAPRLHSPCDAVIYEVHIRDFSIHEDSGMRHKG 240

WP_095240268.1 DPYAKAVTANGEKGVVLDPEEVKVEKHRAPRLHSPCDAVIYEVHIRDFSIHEDSGMRHKG 240

WP_097366433.1 DPYAKAVTANGEKGVVLDPEEVKVEKHRAPRLHSPCDAVIYEVHIRDFSIHEDSGMRHKG 240

WP_101582708.1 DPYAKAVTANGEKGVVLDPEEVKVEKHRAPRLHSPCDAVIYEVHIRDFSIHEDSGMRHKG 240

WP_107661658.1 DPYAKAVTANGEKGVVLDPEEVKVEKHRAPRLHSPCDAVIYEVHIRDFSIHEDSGMRHKG 240

WP_016885603.1 DPYAKAVTANGEKGVVLDPEEVKVEKHRAPRLHSPCDAVIYEVHIRDFSIHEDSGMRHKG 240

********.*******:**:****:*:*** *******:*********************

Pullulanase.FAO.CP7 KYVAFTEDGTETSGGFSTGIAYLKELGVTHIEVLPFHDFARSGCAVSWSQSYNWGYNPLH 255

WP_103750238.1 KYLAFTEDGTETSGGFSTGIAYLKELGVTHIEVLPFHDFAGVD-ELSPDQSYNWGYNPLH 299

WP_048350558.1 KYLAFTEDGTETSGGFSTGIAYLKELGVTHIEVLPFHDFAGVD-ELSPDQSYNWGYNPLH 299

WP_076791128.1 KYLAFTEDGTETSGGFSTGIAYLKELGVTHIEVLPFHDFAGVD-ELAPDQSYNWGYNPIH 299

WP_009329394.1 KYVAFTEDGTETSGGFSTGIAYLKELGVTHIEVLPFHDFAGVD-ELSPDQSYNWGYNPLH 299

WP_095240268.1 KYVAFTEDGTETSGGFSTGIAYLKELGVTHIEVLPFHDFAGVD-ELSPDQSYNWGYNPLH 299

WP_097366433.1 KYVAFTEDGTETSGGFSTGIAYLKELGVTHMEVLPFHDFAGVD-ELSPDQSYNWGYNPLH 299

WP_101582708.1 KYVAFTEDGTETSGGFSTGIAYLKELGVTHIEVLPFHDFAGVD-ELSPDQSYNWGYNPLH 299

WP_107661658.1 KYVAFTEDGTETSGGFSTGIAYLKELGVTHIEVLPFHDFAGVD-ELSPDQSYNWGYNPLH 299

WP_016885603.1 KYVAFTEDGTETSGGFSTGIAYLKELGVTHIEVLPFHDFAGVD-ELSPDQSYNWGYNPLH 299

**:***************************:********* . :: .*********:*

Pullulanase.FAO.CP7 FQCPGRKLFARSTESSKLAQDITESLKTMIQSLPQNTAFSVDYGDVVYNHTYRGKRSRLK 315

WP_103750238.1 FNAPEGSYSLDPQNP----KSRITELKTMIQTLHKH-GFSV-IMDAVYNHVYKRETSPFE 353

WP_048350558.1 FNAPEGSYSLDPQNP----KSRITELKKMIQTLHKH-GFSV-IMDAVYNHVYKRETSPFE 353

WP_076791128.1 FHAPEGSYSLDPQNP----KSRITELKTMIQSLHKH-GFSV-IMDAVYNHVYKRETSPFE 353

WP_009329394.1 FNAPEGSYSLDPQNP----KCRITELKTMIQSLHKH-GFSV-IMDAVYNHVYKRETSPFE 353

WP_095240268.1 FNAPEGSYSLDPQNP----KCRITELKTMIQSLHKH-GFSV-IMDAVYNHVYKRETSPFE 353

WP_097366433.1 FNAPEGSYSLDPQNP----KCRITELKTMIQSLHKH-GFSV-IMDAVYNHVYKRETSPFE 353

WP_101582708.1 FNAPEGSYSLDPQNP----KCRITELKTMIQSLHKH-GFSV-IMDAVYNHVYKRETSPFE 353

WP_107661658.1 FNAPEGSYLLDPQNP----KCRITELKTMIQSLHKH-GFSV-IMDAVYNHVYKRETSPFE 353

WP_016885603.1 FNAPEGSYSLDPQNP----KCRITELKTMIQSLHKH-GFSV-IMDAVYNHVYKRETSPFE 353

*:.* . : : .**.***:* :: .*** *.****.*: : * ::

Pullulanase.FAO.CP7 -NGSRVFFRHNEYGSSTDSTGIENEPVSSSCNGPCCAKIYLDSVRYWLEEYDVDGFRFDL 374

WP_103750238.1 KTVPGYFFRHNEYGFPADGTGVGNDIASE---RLMVRKYILDSVRYWLEEYDVDGIRFDL 410

WP_048350558.1 KTVPGYFFRHNEYGFPADGTGVGNDIASE---RLMVRKYILDSVRYWLEEYDVDGIRFDL 410

WP_076791128.1 KTVPGYFFRHNEYGFPSDGSGVGNDIASE---RLMVRKYILDSVRYWLEEYDIDGIRFDL 410

WP_009329394.1 KTVPGYFFRHNEYGFPSDGTGVGNDIASE---RLMVRKYILDSVRYWLEEYDVDGIRFDL 410

WP_095240268.1 KTVPGYFFRHNEYGFPSDGTGVGNDIASE---RLMVRKYILDSVRYWLEEYDVDGIRFDL 410

WP_097366433.1 KTVPGYFFRHNEYGFPSDGTGVGNDIASE---RLMVRKYILDSVRYWLEEYDVDGIRFDL 410

WP_101582708.1 KTVPGYFFRHNEYGFPSDGTGVGNDIASE---RLMVRKYILDSVRYWLEEYDVDGIRFDL 410

WP_107661658.1 KTVPGYFFRHNEYGFPSDGTGVGNDIASE---RLMVRKYILDSVRYWLEEYDVDGIRFDL 410

WP_016885603.1 KTVPGYFFRHNEYGFPSDGTGVGNDIASE---RLMVRKYILDSVRYWLEEYDVDGIRFDL 410

. ******** :*.:*: *: .*. * ************:**:****

Pullulanase.FAO.CP7 MLYHDIETVRQISTLAENVKPGVYFFGEGWDLNTPLDSGQKATLQNAGKVPAVGFFNDRF 434

WP_103750238.1 MGILDIETVRQISKLAENVKPDALLFGEGWDLNTPLESGQKATLQNAGKVPAVGFFNDRF 470

WP_048350558.1 MGILDIETVRQISKLAENVKPDALLFGEGWDLNTPLESGQKATLQNAGKVPAVGFFNDRF 470

WP_076791128.1 MGILDIETVRQISKLAENVKPGVLLFGEGWDLNTPLESGQKATLQNAGKVPAVGFFNDRF 470

WP_009329394.1 MGILDIETVRQISTLAENVKPGVPLFGEGWDLNTPLDSGQKATLQNAGKVPAVGFFNDRF 470

WP_095240268.1 MGILDIETVRQISTLAENVKPGVLLFGEGWDLNTPLDSGQKATLQNAGKVPAVGFFNDRF 470

WP_097366433.1 MGILDIETVRQISTLAENVKPGVLLFGEGWDLNTPLDSGQKATLQNAGKVPAVGFFNDRF 470

WP_101582708.1 MGILDIETVRQISTLAENVKPGVLLFGEGWDLNTPLDSGQKATLQNAGKVPAVGFFNDRF 470

WP_107661658.1 MGILDIETVRQISTLAENVKPGVLLFGEGWDLNTPLDSGQKATLQNAGKVPAVGFFNDRF 470

WP_016885603.1 MGILDIETVRQISTLAENVKPGVLLFGEGWDLNTPLDSGQKATLQNAGKVPAVGFFNDRF 470

* *********.*******.. :***********:***********************

Pullulanase.FAO.CP7 RNAVKGSTFELSDRGYALGDTGNKAALQHGIAGSPGFLQPAQSINYVSKHDNQTFWDKMA 494

WP_103750238.1 RNAVKGSTFELGDRGYALGDTGKKAELQHGIAGSPGFLLPPQSINYAECHDNHTFWDKMA 530

WP_048350558.1 RNAVKGSTFELGDRGYALGDTGKKAELQHGIAGSPGFLLPPQSINYAECHDNHTFWDKMA 530

WP_076791128.1 RNAVKGSTFELSDRGYALGDTGKKAALQHGIAGSPGFLPPAQSINYVECHDNHTFWDKIA 530

WP_009329394.1 RNAVKGSTFELSDRGYALGDTGKKAALQHGIAGSPGFLQPAQSINYVECHDNHTFWDKMA 530

WP_095240268.1 RNAVKGSTFELSDRGYALGDTGKKAALQHGIAGSPGFLQPAQSINYVECHDNHTFWDKMA 530

WP_097366433.1 RNAVKGSTFELSDRGYALGDTGKKAALQHGIAGSPGFLQPAQSINYVECHDNHTFWDKMA 530

WP_101582708.1 RNAVKGSTFELSDRGYALGDTGKKAALQHGIAGSPGFLQPAQSINYVECHDNHTFWDKMA 530

WP_107661658.1 RNAVKGSTFELSDRGYALGDTGKKAALQHGIAGSPGFLQPAQSINYVECHDNHTFWDKMA 530

WP_016885603.1 RNAVKGSTFELSDRGYALGDTGKKAALQHGIAGSPGFLQPAQSINYVECHDNHTFWDKMA 530

***********.**********:** ************ * *****.. ***:*****:*

Pullulanase.FAO.CP7 LCFKEDADTKRLRQRLAVSIVLLSQGVPFLHAGQEFCRTKNGDSNSYRSGDDINKLDWEK 554

WP_103750238.1 FCSEEDACTKRLRQRLALSIVLLSQGVPFLHAGQEFCRTKNGDSNSYRSGDGINRLDWEK 590

WP_048350558.1 FCSEEDACTKRLRQRLALSIVLLSQGVPFLHAGQEFCRTKNGDSNSYRSGDGINRLDWEK 590

WP_076791128.1 FCSEEDAYSKRLRQRLALSIVLLSQGVPFIHAGQEFCRTKNGDSNSYRSGDDINRLDWEK 590

WP_009329394.1 LCFEEDADTKRLRQRLAVSIVLLSQGVPFLHAGQEFCRTKNGDSNSYRSGDDINKLDWEK 590

WP_095240268.1 LCFKEDADTKRLRQRLAVSIVLLSQGVPFLHAGQEFCRTKNGDSNSYRSGDDINKLDWEK 590

WP_097366433.1 LCFKEDADTKRLRQRLAVSIVLLSQGVPFLHAGQEFCRTKNGDSNSYRSGDDINKLDWEK 590

WP_101582708.1 LCFEEDADTKRLRQRLAVSIVLLSQGVPFLHAGQEFCRTKNGDSNSYRSGDDINKLDWEK 590

WP_107661658.1 LCFEEDADTKRLRQRLAVSIVLLSQGVPFLHAGQEFCRTKNGDSNSYRSGDDINKLDWEK 590

WP_016885603.1 LCFEEDADTKRLRQRLAVSIVLLSQGVPFLHAGQEFCRTKNGDSNSYRSGDDINKLDWEK 590

:* :*** :********:***********:*********************.**:*****

Pullulanase.FAO.CP7 RAELCEDVEYVRQLIRLRRSHPAFRLQKEEEVKEHLSFMDGTGEVTAYKLKNIAAIDPWN 614

WP_103750238.1 RAELCEDVEYVRQLIRLRRSHPAFRLQKEEEVKEHLSFMSGTGEVTAYKLKNIAAFDPWN 650

WP_048350558.1 RAELCEDVEYVRQLIRLRRSHPAFRLQKEEEVKEHLSFMSGTGEVTAYKLKNIAAFDPWN 650

WP_076791128.1 RAELCEDVEYVRQLIRLRRSHPAFRLQKEEEVKEHLSFMSGTGEVAAYKLKNIAAFDPWN 650

WP_009329394.1 RAELCEDVEYVRQLIRLRRSHPAFRLQKEEEVKEHLSFMDGTGEVTAYKLKNIAAIDPWN 650

WP_095240268.1 RAELCEDVEYVRQLIRLRRSHPAFRLQKEEEVKEHLSFMDGTGEVTAYKLKNIAAIDPWN 650

WP_097366433.1 RAELCEDVEYVRQLIRLRRSHPAFRLQKEEEVKEHLSFMDGTGEVTAYKLKNIAAIDPWN 650

WP_101582708.1 RAELCEDVEYVRQLIRLRRSHPAFRLQKEEEVKEHLSFMDGTGEVTAYKLKNIAAIDPWN 650

WP_107661658.1 RAELCEDVEYVRQLIRLRRSHPAFRLQKEEEVKEHLSFMDGTGEVTAYKLKNIAAIDPWN 650

WP_016885603.1 RAELCEDVEYVRQLIRLRRSHPAFRLQKEEEVKEHLSFMDGTGEVTAYKLKNIAAIDPWN 650

***************************************.*****:*********:****

Pullulanase.FAO.CP7 EIIVVHCPFAKKETLKLPDQKQYLLHCDPFTFFNGKVQAEKRLRLNGIGTYVLYEPKG-- 672

WP_103750238.1 EIIVVHCPSAKKETLELPAQKQYLLHCDPFTFFNGKVQAEKRLRLNGIGTYVLYEPKGIF 710

WP_048350558.1 EIIVVHCPSAKKETLELPAQKQYLLHCDPFTFFNGKVQAEKRLRLNGIGTYVLYEPKGIF 710

WP_076791128.1 EIIVVHCPFAKMETLELPDQKQYLLHCDPFTFFNGQVQTEKRLRLNGIGTYVLYEPKGIF 710

WP_009329394.1 EIIVVHCPFAKKETLKLPDQKQYLLHCDPFTFFNGKVQAEKRLRLNGIGTYVLYEPKGIF 710

WP_095240268.1 EIIVVHCPFAKKETLKLPDQKQYLLHCDPFTFFNGKVQAEKRLRLNGIGTYVLYEPKGIF 710

WP_097366433.1 EIIVVHCPFAKKETLKLPDQKQYLLHCDPFTFFNGKVQAEKRLRLNGIGTYVLYEPKGIF 710

WP_101582708.1 EIIVVHCPFAKKETLKLPDQKQYLLHCDPFTFFNGKVQAEKRLRLNGIGTYVLYEPKGIF 710

WP_107661658.1 EIIVVHCPFAKKETLKLPDQKQYLLHCDPFTFFNGKVQAEKRLRLNGIGTYVLYEPKGIF 710

WP_016885603.1 EIIVVHCPFAKKETLKLPDQKQYLLHCDPFTFFNGKVQAEKRLRLNGIGTYVLYEPKGIF 710

******** ** ***:** ****************:**:*******************

Supplementary figure 3. Sequence alignment of the pullulanase catalytic domain, with that of NCBI GenBank pullulanases. The multiple sequence alignment of the amino acid sequence of protein was generated using the software, ClustalW2 of the European Bioinformatics Institute (<http://www.ebi.ac.uk/Tools/msa/clustalw2/>

[1] Cheesbrough, M. (2006) District Laboratory Practice in Tropical Countries. Part 2, 2nd Edition, Cambridge University Press Publication, South Africa, 1-434.

[2] Olutiola P. O., Famurewa, O., Sonntag, H. S. (2000). An Introduction to General Microbiology (A practical Approach). *Measurement of Microbial Growth*, pp. 101-111.

[3] Cheesbrough, M. (2010) District Laboratory Practice in Tropical Countries. 2nd Edition, Cambridge University Press, Cambridge, United Kingdom, 40-127

[4] Ogundolie, F. A. Extracellular Enzymatic Activities of Endophytic Bacteria Isolates Obtained from *Dioclea reflexa* Hook Seeds. Niger. J. Biotechnol., 2024; 41(1), 97-109.

[5] Bhusal, A., & Muriana, P. M. (2021). Isolation and Characterization of Nitrate Reducing Bacteria for Conversion of Vegetable-Derived Nitrate to ‘Natural Nitrite’. Applied Microbiology, 1(1), 11-23. <https://doi.org/10.3390/applmicrobiol1010002>

[6] Ogundolie, F. A. (2021). *Cloning of α-amylase and pullulanase genes of Bacillus licheniformis-FAO. CP7 from cocoa (Theobroma cacao L.) pods and biochemical characterization of the expressed enzymes* (Doctoral dissertation, Doctoral dissertation, Federal University of Technology, Akure). <http://196.220.128.81:8080/xmlui/handle/123456789/4548>

[7] Bisen, P. S. (2014). Laboratory protocols in applied life sciences. CRC Press. Boca Raton, USA, <https://doi.org/10.1201/b16575>
